# Congruence-based contextual plausibility modulates cortical activity during vibrotactile perception in virtual multisensory environments

**DOI:** 10.1038/s42003-022-04318-4

**Published:** 2022-12-12

**Authors:** Kathleen Kang, Robert Rosenkranz, Kaan Karan, Ercan Altinsoy, Shu-Chen Li

**Affiliations:** 1 Chair of Lifespan Developmental Neuroscience, Technische Universität Dresden, Dresden, Germany; 2 Centre for Tactile Internet with Human-in-the-Loop (CeTI), Technische Universität Dresden, Dresden, Germany; 3 Chair of Acoustics and Haptics, Technische Universität Dresden, Dresden, Germany

**Keywords:** Human behaviour, Perception

## Abstract

How congruence cues and congruence-based expectations may together shape perception in virtual reality (VR) still need to be unravelled. We linked the concept of plausibility used in VR research with congruence-based modulation by assessing brain responses while participants experienced vehicle riding experiences in VR scenarios. Perceptual plausibility was manipulated by sensory congruence, with multisensory stimulations confirming with common expectations of road scenes being plausible. We hypothesized that plausible scenarios would elicit greater cortical responses. The results showed that: (i) vibrotactile stimulations at expected intensities, given embedded audio-visual information, engaged greater cortical activities in frontal and sensorimotor regions; (ii) weaker plausible stimulations resulted in greater responses in the sensorimotor cortex than stronger but implausible stimulations; (iii) frontal activities under plausible scenarios negatively correlated with plausibility violation costs in the sensorimotor cortex. These results potentially indicate frontal regulation of sensory processing and extend previous evidence of contextual modulation to the tactile sense.

## Abstract (web summary)

The effect of trimodal visuo-audio-tactile stimulations on perceptual realism during vehicle riding simulations in virtual reality reveals that multisensory plausibility engages cortical activities in the frontal-parietal and sensorimotor regions.

## Introduction

Human behaviours are embodied through the senses and embedded in a system of contexts^1,2^. Moreover, humans also actively construct and select contexts for their behaviours and adapt to them^3,4^. Thus, other than the physical and social environments, breakthroughs in computer science as well as in communication and other digital technologies in the past decades have created new digital environments for human behaviours and the research about them. Specifically, virtual reality (VR) technologies offer a broad spectrum of experiential contexts that are applicable in many domains, ranging from industry to education and medicine^5,6^. Within the communities of psychological and cognitive neuroscience research, VR technologies have been increasingly used in lab-based experiments to enable more naturalistic studies of human behaviours^7–10^. There are, however, still considerable concerns about the discrepancies between experiences in the real and virtual worlds^11–14^. Such gaps arise, in part, from the still rather limited use of multisensory inputs in VR, which usually do not go beyond vision and hearing.

Albeit its widespread usages, most VR applications rely only on visual and auditory information^15^. The inclusion of other sensory modalities, such as touch, into VR technologies are currently being developed and evaluated^15,16^. Towards this goal, recent developments in digital, telecommunication, as well as sensor and actuator technologies have joined force to establish a type of digital communication infrastructure, known as the *Tactile Internet*^17–19^, for humans to remotely access, perceive, and manipulate real or virtual objects. These technologies could provide multisensory avenues for humans to experience virtual (or remote) environments through digitalized tactile and kinesthetic information, besides visual and auditory signals. The tactile sense operates with a more precise temporal resolution^20^ and develops earlier in life than hearing and vision^21^. Therefore, the inclusion of tactile information could further enhance multisensory perception in virtual environments. En route to developing digital infrastructures for humans to behave in virtual or remote settings, it is crucial to understand neurocognitive mechanisms underlying multisensory perception^22,23^. Thus, we investigated cortical processes for the combined influence of bottom-up sensory congruence cues and top-down experience-based expectations on tactile perception in virtual multisensory environments.

In VR research, the plausibility of external events in the virtual environment is considered as one of the key factors for constructing virtual experiences that could be perceived as sufficiently realistic^24,25^. Plausibility in this regard refers to correspondences between sensory events in VR. Furthermore, the credibility of plausible external events can be maintained if they confirm with what normally would be expected in the rendered circumstances (cf. 24). This notion of perceived perceptual plausibility in VR being affirmed through a conformation with what would be expected in normal circumstances is in line with Helmholtz’s classical view (1857)^26^ and the more recent Bayesian approaches of perception^2,27–29^. According to these theories, perception is guided, in part, by expectations that are based on the individual’s past experiences or prior knowledge. Such top-down influences act together with bottom-up congruency cues that are driven by low-level stimulus properties (e.g., spatial or cross-modal congruency) for humans to form coherent and robust multisensory perception^27–32^. Regarding brain substrates for such processes, the inferior frontal lobe plays a role in combining top-down congruency expectations with bottom-up congruency cues to adjust information integration and segregation during multisensory perception^29^. A general aim of this study is to experimentally relate the concept of plausibility used in developing VR technologies with neurocognitive studies of multisensory perception to facilitate interdisciplinary research.

Thus far, studies on virtual realism have primarily evaluated the plausibility of external events in VR by subjective ratings^33,34^ or behavioural measures^35^. However, subjective ratings are known for their methodological limitations on validity^34^. Besides, behavioural observations alone could not elucidate neurocognitive processes involved in the interplay between sensory congruence and contextual expectation that together may affect the perceived plausibility of multisensory experiences in VR. Knowledge about these processes can shed light on how congruence-based contextual plausibility may modulate multisensory perception in more naturalistic settings and inform engineering solutions for plausible virtual multisensory experiences.

Empirical findings from primate and human studies have identified several relevant brain areas for tactile and multisensory perception. For instance, neuronal activity recorded in awake monkeys trained to detect vibrotactile stimuli showed that neuronal representations associated with vibrotactile stimulations unfold in time across several cortical regions, ranging from the earlier somatosensory cortex over the motor cortex to the later dorsal and ventral premotor cortex^36,37^. Moreover, in situations with substantial sensory uncertainty, such as when processing near-threshold vibrotactile stimuli, the subjective experience of signal detection was found to be correlated with activities in the frontal regions (e.g., premotor cortex) known to support top-down attentional control and action planning^36^. Besides, the parietal cortex also plays important roles in multisensory perception of trimodal stimuli which encompasses vision, hearing, and touch^38^. Furthermore, results from human studies of visual-auditory illusions^39^ showed that brain activities in the inferior frontal lobe contribute to incongruency effects when bottom-up sensory congruency cues conflicted with top-down contextual expectations^29,40^.

Along with the aforementioned general goal, our specific aim is to investigate how perceived perceptual plausibility that is jointly influenced by sensory congruence cues and congruency expectations may modulate cortical activities in frontal and sensorimotor regions, as well as their relations during vibrotactile perception in virtual multisensory environments. Furthermore, we were also interested in finding out whether congruence-based expectation would directly impact cortical responses in the sensorimotor cortex, beyond the effects of vibrotactile stimulation intensity. Previous studies showed that the effects of congruence-based expectation on perception can either be facilitative or inhibitory, depending on task-specific demands or response strategies^41,42^. Whereas task-specific response strategies may reflect perceptual decision biases, multisensory tasks without explicit response requirements are more suitable in capturing automatic perceptual processes that occur in many real-life situations^41,43^.

Regarding the first question, previous findings from studies on prior expectations based on semantic contexts showed that, when no explicit perceptual decision or response are involved, perceptual processing favours sensory inputs that are congruent (confirmed) over those that are incongruent (disconfirmed) with what would be expected based on prior experiences or knowledge^31,44^, and engages more neural activities in the frontal^45^ and sensory^41^ cortices. We thus hypothesized greater hemodynamic responses during the high in comparison to the low plausibility scenarios. As for the second question, past studies also indicated that congruence-based expectations could induce substantial changes in perceptual representations beyond just biasing perceptual selections. Such effects have previously been shown in audio-visual^27^, taste^46,47^, and pain^48^. We, therefore, also expected that the responses of the sensorimotor cortex to vibrotactile stimulations of a given intensity would be modulated by multisensory contextual congruency in the virtual environment. Specifically, we hypothesized that experiencing a weaker vibrotactile stimulation may nonetheless elicit a larger response in the sensorimotor cortex than a stronger stimulation, if the vibrotactile signal of a lower intensity would be more in line with the participant’s congruency expectation given by the audio-visual information embedded in the virtual scenario. We also explored the relation between the effects of congruence-based plausibility on frontal cortical responses and subjective ratings of vibrotactile stimulation plausibility.

To test these hypotheses, we created several virtual scenarios of front-row vehicle riding to investigate the underlying neurocognitive mechanisms. The plausibility level of vibrotactile stimulations was experimentally operationalized by manipulating the congruence (match) or incongruence (mismatch) between stimulation intensity and the contextual audio-visual information of different virtual vehicle riding scenarios. The daily experiences of being a passenger riding in a car that moves through roads with different surface conditions are common for adults. Acquired prior knowledge that is based on such common experiences serves as a basis for expecting weaker vibrations when riding on smooth roads and stronger vibrations when confronted with rough road situations. In this way, the sensory (in)congruence between vibrotactile stimulation intensity and the audio-visual information embedded in the virtual scenarios of moving through roads of different surface types allowed us to vary the degrees of conformity between the experienced multisensory congruence cues in VR and the participant’s expectations about vibrations they would normally expect for a given road type.

Specifically, in each of the virtual scenarios, a vibrotactile stimulation of high (e.g., 36 dB above perceptual threshold) or low (e.g.,10 dB above perceptual threshold) intensity from a car seat was presented concurrently with the audio-visual information for a given road scene. The vibration was delivered through the seat that was securely mounted on a hydraulic platform with an electrodynamic shaker. The scenes displayed views from the front passenger row of a vehicle moving through rougher (e.g., cobblestone) or smoother (highway) road surfaces that are, respectively, accompanied by congruent louder or quieter audio sound effects recorded from the corresponding situations (see “Methods” for details). The road scenes were carefully selected to be representative of daily experiences. Thus, when participants were exposed to the audio-visual contexts of the various scenarios, they would have general expectations about the vibration strengths to be felt from the seat, which would normally be associated with the respective audio-visual information of the road scenes. The different intensity levels of vibrotactile stimulations were crossed with the audio-visual information for the different road scenes, which allowed us to vary street scene-based contextual congruency expectation^29^ about the vibrotactile experience for each of the scenarios. Accordingly, the experiment included virtual scenarios of high plausibility (i.e., vibrotactile intensity confirmed with the contextual expectation given by the corresponding audio-visual information displayed for a road scene) or low plausibility (vibrotactile intensity disconfirmed with the contextual expectation).

We measured brain hemodynamic responses using functional Near-Infrared Spectroscopy (fNIRS) while human participants were exposed to the virtual scenarios of vehicle riding. Given previous findings on brain regions relevant for vibrotactile and trimodal perception, we used a montage with the NIRS sources and detectors covering the dorsolateral prefrontal (dlPFC), premotor (including the supplementary motor area) and the sensorimotor regions. While being on the virtual rides, the participants were asked to simply experience the situations and reflect about the plausibility of the vibrations felt through the seat. No explicit responses were required during the main experiment. Besides the main experiment, subjective plausibility ratings of vibrotactile stimulations over a wider range of intensity levels were also obtained from the participants (see “Methods” for details).

To anticipate, results of the present study indicate that plausible vibrotactile stimulations which conformed to the participants’ congruency expectations that are probed by the contextual audio-visual information embedded in the virtual scenarios elicited greater brain hemodynamic responses than stimulations of low plausibility. Notably, other than engaging a higher level of frontal cortical activity, congruence-based expectations about the intensities of vibrotactile stimulation also impacted perceptual representations in the sensorimotor cortex. Moreover, we observed negative relations between individual differences in frontal activities observed during high plausibility scenarios and the congruence-based plausibility violation costs measured in the sensorimotor cortex.

## Results

### Higher cortical activities in scenarios with high congruence-based plausibility

**Fig. 1:**
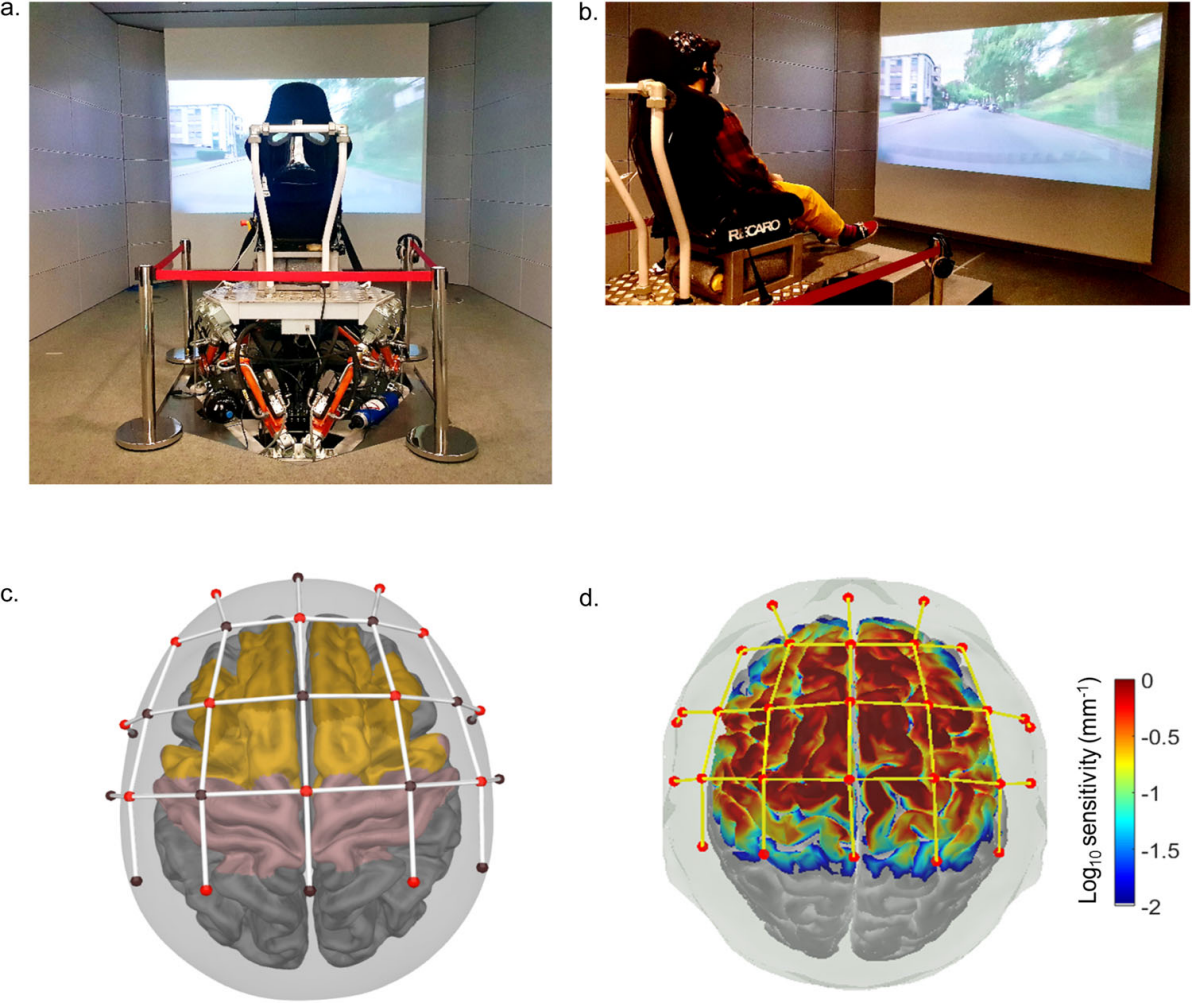
Experimental setup of the multisensory virtual car riding environment and NIRS montage. **a** Vertical vibrotactile stimulation was displayed through a seat mounted on a hydraulic platform and an electrodynamic shaker. **b** Videos of moving road scenes were displayed on a wall-sized screen together with the corresponding audio recordings reproduced with a wavefield synthesis system with 464 loudspeakers in the room to provide contextual visual-auditory information for the vibrotactile stimulation. A participant wearing the NIRS cap sat on a seat in the virtual environment during the experiment. **c** The layout of the NIRS montage used in this study covered the dorsal lateral prefrontal cortex (dlPFC, dark yellow), premotor cortex with supplementary motor area (light yellow), primary motor, and primary somatosensory cortex (pink). **d** The sensitivity profile of the montage indicates high sensitivity to optical density changes measured by each of the source-detector pairs in brain regions of (values shown here are on the log_10_ scale of sensitivity values from 0.01 to 1.0).

We first examined the impact of congruence-based plausibility on cortical activities when participants passively experienced virtual scenarios with audio-visual scenes of roads (e.g., see Fig. 1a, b) of different surface roughness (i.e., cobblestone, fine cobblestone, tarmac and highway roads) that were combined with vibrotactile stimulations of different intensities (the videos, sound files, and digital files of high as well as low plausibility vibrotactile stimulations for the virtual scenarios are available through the link provided in the “Data availability” statement). This manipulation jointly varied bottom-up multisensory congruence cues and top-down congruence expectations, thus yielding scenarios of high or low plausibility, depending on congruency or incongruency between the audio-visual scenes and the intensities of vibrotactile stimulations.

The participants’ subjective plausibility ratings of vibrations across a larger range of intensity levels were obtained from a separate phase of the study. These ratings confirmed that the vibrotactile stimulations used in the high plausibility scenarios were rated as more plausible (mean ratings ranged from 59.93 to 79.12) than those used in the low plausibility scenarios (mean ratings ranged from 4.46 to 56.05), *F*(1, 290) = 280.35, *p* < 0.001, *η*_*p*_^*2*^ = 0.49.

**Fig. 2:**
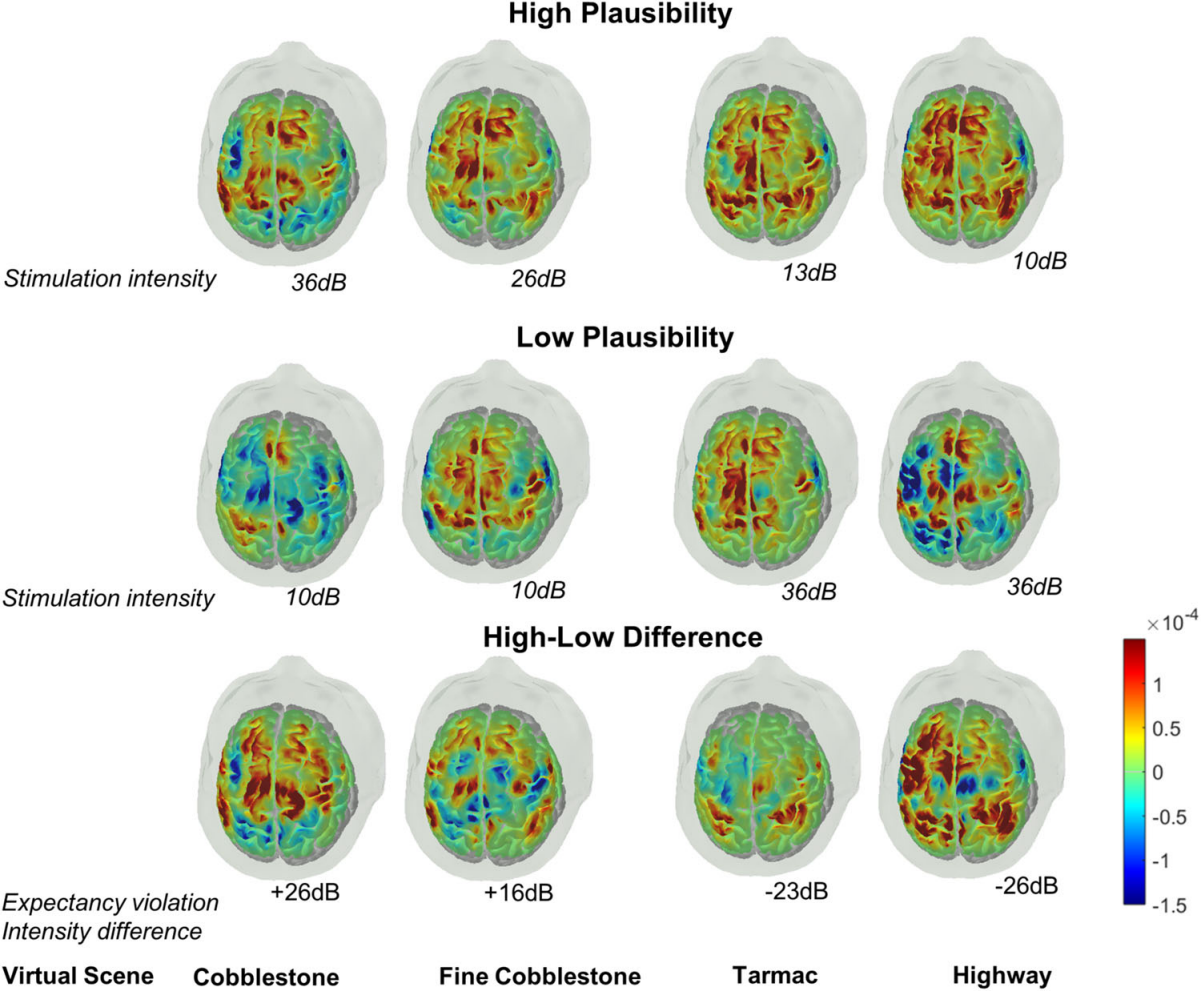
Topographical plots of levels of HbO concentration across all channels. The different columns correspond to the four different road scenes. The top and middle rows show results, respectively, from scenarios with vibrotactile stimulations at intensity levels of higher and low plausibility. The third row shows contrast plots (high–low plausibility).

We used linear mixed-effects models to analyze data collected from young adult participants (*N* = 36), with experimental factors (road scene and plausibility) as fixed effects, and random intercepts for participants with NIRS channels nested in participants (the relatively large number of observations from each participant, i.e., on average 36 NIRS channels for each of the 8 scenarios resulted in large degrees of freedom that influenced the calculation of effect sizes^49^; see “Methods” for detailed descriptions of the participants, effective sample size, and further information about the linear mixed-effects models). Results from the overall analysis of concentration levels of oxygenated haemoglobin (HbO) measured from all NIRS channels (see “Methods” for procedures of data pre-processing and motion artifact correction) revealed significant main effects of road scene (*F* (3, 8617) = 12.79, *P* < 0.0001, *η*_*p*_^*2*^ = 0.004, and plausibility (*F* (1, 8617) = 79.30, *P* < 0.0001, *η*_*p*_^*2*^ = 0.009). This pattern of results indicates that the levels of HbO concentration differed between the virtual scenarios as a function of contextual expectancy (Fig. 2). Across scenes of all road types, HbO concentration was higher in virtual scenarios of high than low plausibility, suggesting contextual expectancy modulates cortical activity. Of note, these effects were specific for HbO concentration and were not observed for deoxygenated haemoglobin (HbR; *P* = 0.18, *η*_*p*_^*2*^ = 0.0006 and *P* = 0.17, *η*_*p*_^*2*^ = 0.0002 for the main effects of road scene and plausibility, respectively). Furthermore, previous research also showed that, changes in HbO levels are more associated with task-induced cortical responses than HbR concentration levels^50^ (see also further information in “Methods”). Thus, below we only focus on results based on HbO. Besides the main effects, the scene by plausibility interaction was also significant (*F* (3, 8617) = 7.93, *P* < 0.0001, *η*_*p*_^*2*^ = 0.003), indicating that the effect of contextual expectancy on HbO concentration was larger in scenes with extreme roughness (cobblestone, *t* (8617) = 5.26, 95% CI [0.000002 0.000007], *P* < 0.0001, *d* = 0.21) or extreme smooth surface (highway, *t* (8617) = 8.09, 95% CI [0.000004 0.0000096], *P* < 0.0001, *d* = 0.33).

### Plausibility modulation of cortical activities in frontal and sensorimotor regions

**Fig. 3:**
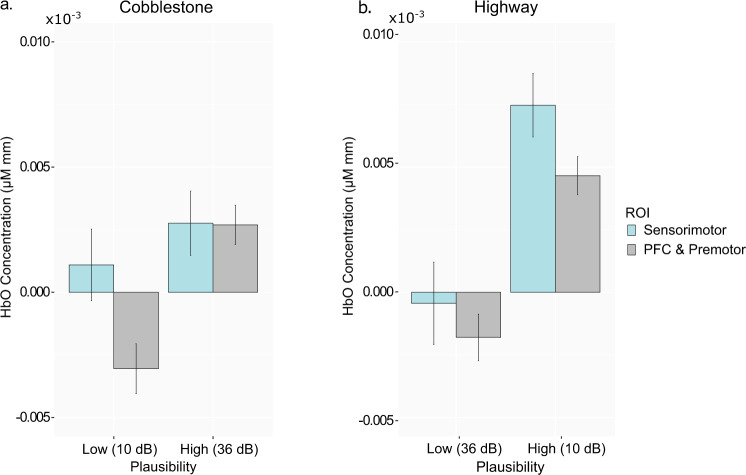
Averaged HbO concentration in the frontal regions and somatosensory cortex. **a** Shown here are effects of congruence-based plausibility and cortical regions for the scenes of cobblestone road and **b** Shown here are effects of congruence-based plausibility and cortical regions for the scenes of highway. Error bars represent ± 1 standard error of mean.

Following up the results across all NIRS channels and road scenes, we next conducted separate analyses for the scenes with extreme roughness (cobblestone) or smoothness (highway), which also had the largest intensity difference (26 dB) between high and low plausibility conditions. We compared HbO concentrations in the sensorimotor cortex with those in the dorsolateral prefrontal and premotor regions, which are known to be involved in processes of cognitive control, planning, and conceptual expectations^45,51,52^ as well as in combined effects of top-down congruency expectation and bottom-up congruence cues during multisensory perception^29,40^. The results (Fig. 3) again revealed the main effect of plausibility in each of the two scenarios (cobblestone: *F* (1, 1230) = 22.50, *P* < 0.0001, *η*_*p*_^*2*^ = 0.02; highway: *F* (1, 1230) = 52.68, *P* < 0.0001, *η*_*p*_^*2*^ = 0.04). The main effect of brain regions (sensorimotor vs. dlPFC & premotor) was also significant in each of the two analyses: the HbO concentration was higher in the sensorimotor cortex than in the frontal regions (cobblestone: *F* (1, 1195) = 4.92, *P* = 0.027, *η*_*p*_^*2*^ = 0.004; highway: *F* (1, 1195) = 4.48, *P* = 0.03, *η*_*p*_^*2*^ = *0.004*). The brain region × plausibility interaction was significant only in the road scene with extreme roughness (cobblestone: *F* (1, 1230) = 3.95, *P* = 0.047, *η*_*p*_^*2*^ = 0.003; highway: *F* (1, 1230) = 0.49, *P* = 0.48, *η*_*p*_^*2*^ = 0.0004), due to the relative lower HbO concentration level in the frontal regions in the low plausibility scenario in the cobblestone scene.

Plausibility in the current study was experimentally manipulated by crossing scenes of rough (cobblestone) or smooth (highway) roads with vibrotactile stimulations of high (36 dB above perceptual threshold) or low (10 dB above perceptual threshold) vibration intensities. This allowed us to conduct further follow-up analyses to examine the nature of the effects of contextual expectancy. First, we compared effects of vibrotactile stimulation of a given intensity in the sensorimotor cortex between scenes (highway vs. cobblestone). On the one hand, when contrasting HbO concentration in the sensorimotor cortex under the 10 dB stimulation in the cobblestone scene with that in the highway scene (cobblestone_10dB_ − highway_10dB_), we observed a significantly lower HbO level of the same stimulation intensity (*t* (1131) = −3.48, 95% CI [−0.000011 −0.0000015], *P* = 0.0005, *(Bonferroni-)Adjusted P* = 0.003*, d* = *−*0.23*)* in the scene with lower multisensory plausibility. For the scene with the rough road surface (i.e., cobblestone), this difference reflected an effect of negative expectancy violation cost, since 10 dB fell short of the expectation implied by the audio-visual information in the virtual scenario of the cobblestone road. On the other hand, when contrasting HbO concentration in the sensorimotor cortex under the 36 dB stimulation in the cobblestone scene with that in the highway scene (cobblestone_36dB_ − highway_36dB_), we observed a marginally higher HbO level of the same stimulation intensity (*t* (1131) = 1.744, 95% CI [−0.0000017 0.0000080], *P* = *0.08, Adjusted P* = 0.488*, d* = 0.12*)* in the scene with higher multisensory plausibility. For the scene with smooth road, this reflected an effect of positive expectancy violation cost, since 36 dB surpassed the expectation implied by the audio-visual contextual information in the virtual scenario of the highway).

We then compared effects of vibrotactile stimulations of different intensities in the same scene as a function of expectation congruence or violation. In the scene of smooth road (highway), a lower intensity (10 dB) of vibrotactile stimulation that was congruent with the expectation given by the audio-visual information of the scene elicited a higher HbO level in the sensorimotor cortex than a stronger (36 dB) vibrotactile stimulation that violated the contextual expectation (*t* (1230) *=* 4.61, 95% CI [0.0000034 0.0000124]*, P* < *0.0001, Adjusted P* < 0.0001*, d* = 0.30). An analysis of HbO levels associated with the same scene (highway) in the frontal regions revealed a similar effect (*t* (1230) *=* 5.65, 95% CI [0.0000034 0.0000095]*, P* < 0.0001*, Adjusted P* < 0.0001*, d* = 0.25). In the scene of rough road (cobblestone), similar patterns of results were only observed in the frontal regions (*t* (1230) *=* 5.05, 95% CI [0.0000027 0.0000088]*, P* < 0.0001*, Adjusted P* < 0.0001*, d* = 0.22), but not in the sensorimotor cortex (*t* (1230) = 0.97, 95% CI [−0.0000029 0.0000062], *P* = 0.33, *Adjusted P* > 0.99, *d* = 0.06).

#### Congruence-based plausibility modulates frontal-somatosensory associations

As a next step, we conducted correlational analyses to examine how congruence-based plausibility may modulate functional relations between cortical activities in the frontal and sensorimotor regions during multisensory perceptual experiences in VR scenarios. Given that individual differences in the signal-to-noise ratios of the NIRS signals may confound between-person correlations, baseline correction was performed for each participant (see “Methods” for details). Furthermore, we computed plausibility violation cost scores for each of the participants, based on the signal amplitude of an individual’s cortical activities in the low plausibility condition as his or her own control. Lastly, we also conducted a further control analysis by checking correlations based on ratio scores for each participant (i.e., normalizing plausibility violation costs through dividing them by the means of cortical activities of both conditions at the individual level). Since the ratio scores were not normally distributed, we conducted Spearman’s correlation for the ratio scores.

**Fig. 4:**
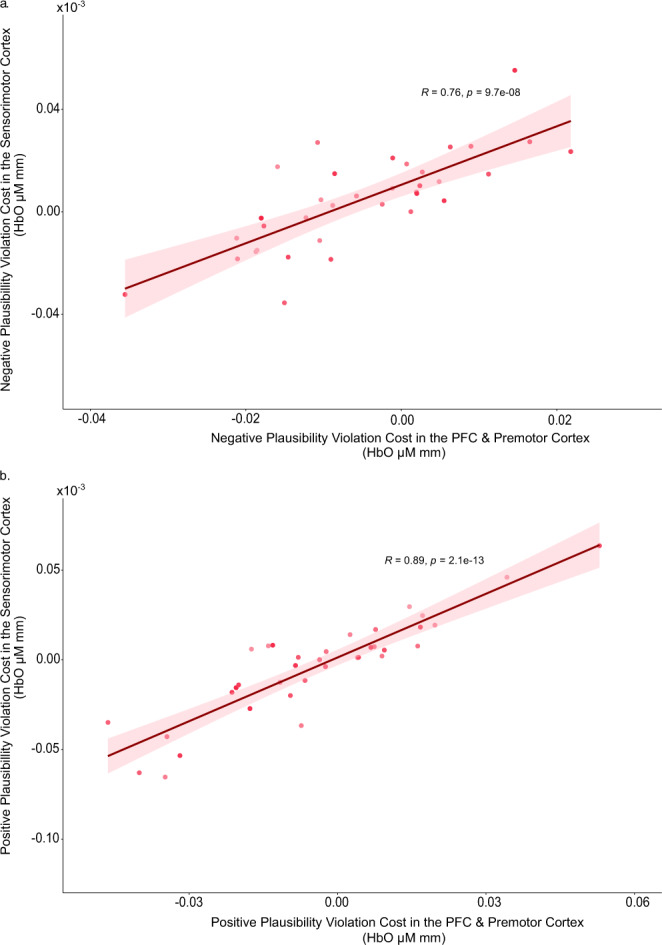
Scatterplots showing positive correlations between plausibility violation costs in the somatosensory cortex and frontal regions. **a** Results regarding negative expectancy cost which was computed as the difference in HbO concentration between the scenarios of cobblestone_10dB_ and highway_10dB_ (*n* = 35 young adults). **b** Results regarding positive expectancy cost which was computed as the difference in HbO concentration between the scenarios of highway_36dB_ and cobblestone_36dB._ Shaded area represents the 95% confidence interval (*n* = 36 young adults).

We first correlated the magnitudes of expectancy modulation of HbO concentrations in the frontal regions with those in the sensorimotor cortex separately for the modulation effects involving negative or positive expectancy violations. Independent of valence (negative or positive), greater magnitudes of expectancy modulation in the frontal regions are highly correlated with greater modulation effects in the sensorimotor region across individuals (Fig. 4a: negative plausibility violation cost: *r*_cost_ (33) = 0.76, *P* < 0.0001, *N* = 35; Fig. 4b: positive plausibility violation cost: *r*_cost_ (34) = 0.89, *P* < 0.0001, *N* = 36). Control analyses computed based on ratio scores that were normalized by individual means of HbO concentrations of both plausibility conditions also yielded significant, albeit attenuated, effects (negative: *rho*_ratio_ (33) = 0.68, *P* < 0.0001, *N* = 35; positive: *rho*_ratio_ (32) = 0.43, *P* = 0.01, *N* = 34). These results indicate that congruence-based plausibility systematically modulates cortical activities in the frontal and sensorimotor regions.

**Fig. 5:**
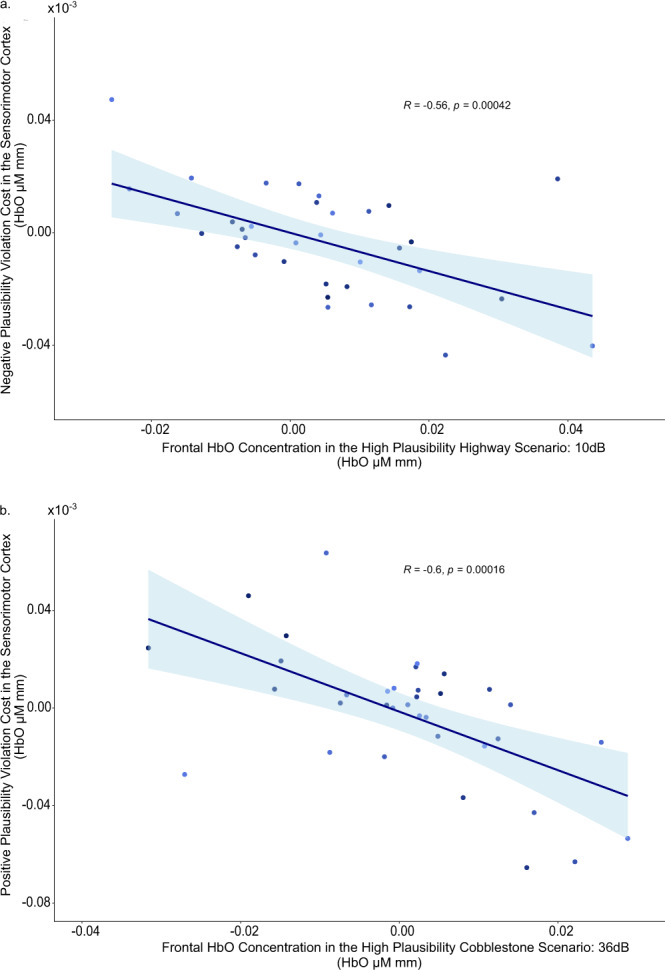
Scatterplots showing negative correlations between plausibility violation costs in the somatosensory cortex and HbO concentration in the frontal regions. **a** Results regarding the scenario of high plausibility with 10 dB vibrotactile stimulation (*n* = 35 young adults). **b** Results regarding the scenario of high plausibility with 36 dB vibrotactile stimulation. Shaded area represents the 95% confidence interval (*n* = 35 young adults).

Next, we examined the relations between levels of HbO in the frontal regions under scenarios of high multisensory plausibility (i.e., the scenes with highway or cobblestone road that were paired with the 10 dB or 36 dB vibrotactile stimulations, respectively) and the magnitudes of expectancy violation costs in the sensorimotor cortex. Again, independent of the valence of expectancy violation, we observed negative correlations between levels of HbO concentration in the dlPFC and premotor regions under high plausibility scenarios and the magnitude of expectancy violation costs in the sensorimotor cortex (Fig. 5a: high plausibility virtual scenario with 10 dB stimulation, *r*_cost_ (33) = −0.56, *P* < 0.001, *N* = 35; Fig. 5b: high plausibility virtual scenario with 36 dB stimulation, *r*_cost_ (33) = −0.60, *P* < 0.001, *N* = 35). Validating these results by control analyses computed based on ratio scores also yielded comparable significant effects (10 dB stimulation: *rho*_ratio_ (33) = −0.55, *P* < 0.001, *N* = 35; 36 dB stimulation: *rho*_ratio_ (31) = −0.75, *P* < 0.001, *N* = 33). These correlations indicate that individual differences in engaging activities in the dlPFC and premotor cortex when processing contextually congruent multisensory information are negatively associated with individual differences in recruiting cortical activities in sensorimotor cortex when contextual expectations were violated.

**Fig. 6:**
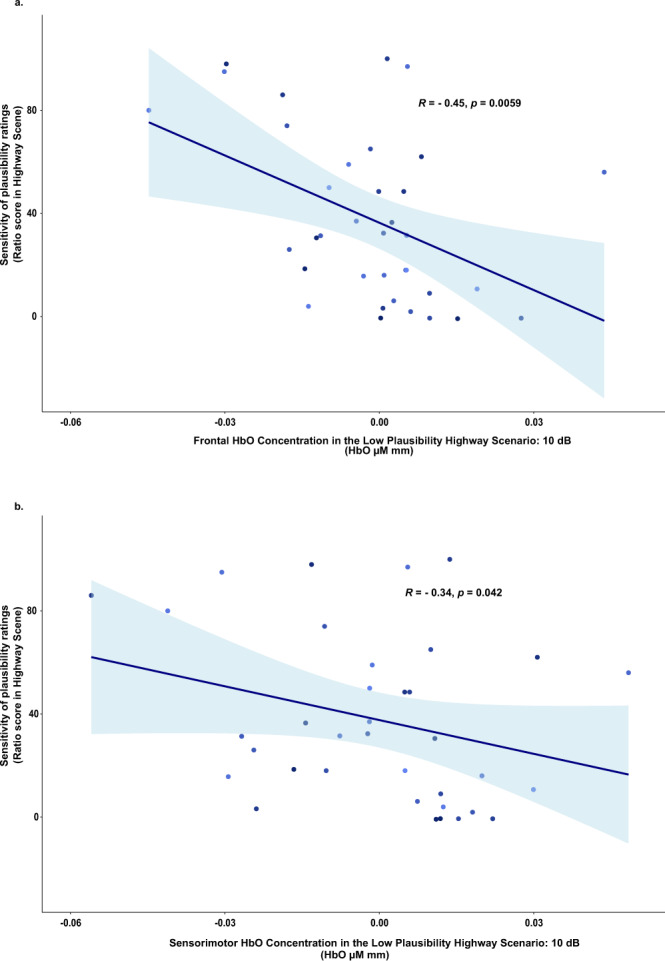
Scatterplots showing negative correlations between cortical activities under the low plausibility scenario and the sensitivity of subjective ratings of perceived plausibility. **a** Results showing HbO concentration in the low plausibility scenario with smooth road surface (highway) in the frontal and premotor regions (*n* = 35 young adults). **b** Results showing HbO concentration in the low plausibility scenario with smooth road surface (highway) in the sensorimotor cortex (*n* = 35 young adults). Shaded area represents the 95% confidence interval.

Lastly, as an exploratory analysis we correlated individual subjective ratings of perceived plausibility of the vibrotactile stimulations that were collected in addition to the main experimental task separately with HbO concentrations in the frontal regions and in the sensorimotor cortex. Significant negative correlations between individual differences in the sensitivity of subjective ratings (i.e., the difference between ratings of high vs. low plausibility scenarios divided by the rating of low plausibility scenario) were observed in the frontal regions (*rho* (34) = −0.45, *P* = 0.006, *N* = 36; see Fig. 6a) and in the sensorimotor cortex (*rho* (34) = −0.34, *P* = 0.04, *N* = 36; see Fig. 6b) in the low plausibility scenario with the scene of smooth road surface (highway). No such correlations were observed for the high plausibility scenario with the highway scene or scenarios with the rougher surfaces.

## Discussion

Combining a naturalistic setup for vehicle riding experiences in virtual multisensory scenarios with assessing brain hemodynamic responses using fNIRS, we identified brain mechanisms associated with the plausibility principle of virtual realism^24,25^, which operated through multisensory congruence cues and congruency expectations^26–29,53^ that were contextually embedded in the virtual environments. Congruency between the experienced intensity of vibrotactile stimulations and the audio-visual information available in the virtual scenarios that also confirmed with the individual’s contextual expectation based on prior experiences with similar road types render more plausible perception in VR. Such confirmed congruence-based plausibility engaged greater activities in cortical regions (dlPFC and premotor cortex) which are important for top-down congruency expectation modulation and planning of cognition and behaviour^54^, as well as activities in the sensorimotor cortex implicating sensory and motor processing^55^. Of note, independent of the actual stimulation intensity, greater cortical activities were observed in the sensorimotor cortex for vibrotactile events with congruent audio-visual sensory cues and congruent road scene-based expectations. These results reveal that congruence-based contextual plausibility modulates vibrotactile perceptual representations in the sensorimotor region (see Figs. 2 and 3). Furthermore, negative correlations between individual differences in frontal activities under virtual scenarios supporting multisensory expectancy confirmation and the magnitude of plausibility violation costs in the sensorimotor cortex (see Fig. 5) indicates that those individuals who were more sensitive in engaging frontal activities during confirmed multisensory contextual expectations also yielded weaker perceptual representations in the sensorimotor cortex when the experienced vibrotactile stimulations were of low congruence-based plausibility. Results from the exploratory analyses revealed correlations between cortical activities and individual differences in subjective ratings of perceptual plausibility. Individuals who showed a greater sensitivity in subjective plausibility ratings also engaged weaker frontal and sensorimotor activities when the sensory events experienced in the VR environment were low in plausibility. This finding, however, was only observed for the road scene with smooth surface, indicating that while subjective ratings may be related with underlying neurocognitive processes, they are less discriminant for individual differences in ratings under conditions of high plausibility scenarios or rough road surface where the normally expected stimulation intensities may tend to be high.

Taken together, findings from this study empirically link the construct of plausibility in the research on the design and evaluation of VR technologies^24,25^ with established psychological and neurocognitive research on mechanisms and effects of expectations on multisensory perception^27–29,53^. However, whether current results can be generalized to more immersive settings that use head-mounted displays in VR space with visual projections on several walls (known as CAVE technologies) still need to be investigated in future research. Whereas many previous studies on brain mechanisms of expectancy modulation have focused mainly on auditory and visual modalities^29,31,41,44,45^, the findings of the present study extend these mechanisms to the tactile sense. This study also lends further support for the Bayesian inference framework of embodied and contextually embedded perception^2,27–29^, which formalizes contextual expectations as statistical priors about the properties of the multisensory environment during perception and action.

Evidence from previous studies that used auditory-visual illusions^39^ to investigate mechanisms of expectations on multisensory perception shows that blood-oxygen-level-dependent (BOLD) responses in the inferior frontal gyrus^40^ and inferior frontal sulcus^29^ are sensitive to sensory congruency. The current results of confirmed contextual expectations modulating HbO concentrations in the frontal and sensorimotor regions are only partly consistent with the earlier evidence, since instead of an incongruency-related upregulation of cortical activities, here we observed increased HbO responses during conditions of confirmed contextual expectations. It is important to note, however, that perceptual illusion tasks usually involve competing responses (illusory or non-illusory percept). The potential perceptual and response conflicts in the non-congruent conditions engaged more, instead of less, frontal activities relative to the congruent conditions in such settings. Thus, depending on task-specific demands and response strategies, sensory stimulations that are either congruent or incongruent with expectations could both trigger greater brain responses^41,42^. Our finding that vibrotactile stimulations congruent with the expectations given by the audio-visual information engaged greater cortical activities is in good agreement with previous results, further reiterating that passive perceptual processing favours congruent over incongruent information^29,44^ and engages more neural activities in the frontal^45^ and sensory^41^ cortices. Furthermore, previous research on visual scene contextual facilitation of object perception yielded findings that are in line with the direction of the effects observed here. Neural responses in the visual cortex during visual object processing were found to be enhanced by embedding objects in expected visual scenes^56^.

Other than the effects on frontal regions, we also observed that the levels of HbO concentrations in the sensorimotor cortex were higher when the intensities of vibrotactile stimulations felt through the seat were as expected based on the audio-visual information in the virtual environments. Moreover, the plausibility-related upregulation of activities in sensorimotor cortex was positively correlated with plausibility-enhancing effects in the frontal and premotor regions. This finding parallels with a previous result from the visual modality, which revealed correlated cortical effects of scene contextual facilitation in brain regions of visual object processing and in the areas of higher-level expectation-derived scene processing, including the  parahippocampal place area and the retrosplenial cortex^56^. The mere intensity of the stimulation did not directly affect perceptual representations in the sensorimotor cortex. A stronger stimulation level (36 dB that violated the contextual expectation associated with riding over a smooth road given the audio-visual information in the virtual scenario of a highway resulted in a 7-fold weaker response in the sensorimotor cortex compared to the expected weaker stimulation (10 dB). This result extends previous findings on the powerful effects of contextual congruency in changing perceptual representations that were found in auditory, visual, taste, and pain perception^27,46–48^.

The role of the prefrontal cortex in flexibly gating sensory processing for context-dependent or goal-directed behaviour has been well established in animal and human studies^57^. Here, the observed relationship between frontal activities during virtual scenarios with vibration intensities that confirmed with contextual expectations and the expectancy violation costs as reduced responses in the sensorimotor cortex are in line with previous research. There are anatomical and functional connectivity for frontal regulations of sensory processing. For instance, frontal regulations of sensorimotor processing could be channelled through the cortico-cortical pathway, starting from the dlPFC, premotor cortex, supplementary motor area to the sensorimotor cortex and parietal regions^58^. Although the PFC does not project directly to the modality-specific sensory regions in the thalamus, it may filter sensory signals indirectly through regulating the basal-ganglia-thalamus pathways^59^. Early human brain lesion research indicates that the prefrontal cortex may gate somatosensory inputs through frontal-parietal pathway, as demonstrated by a reduction in somatosensory evoked potentials observed in patients with PFC lesions^60^. Using non-invasive brain stimulation technique, a recent study also showed that high-frequency repetitive transcranial stimulation over the dlPFC modulates sensorimotor cortex’s adaption to pain perception^61^.

Thus far, the interdisciplinary exchanges between cognitive neuroscience and virtual reality (VR) technologies have been mainly focusing on using VR as a means to enhance ecological and dynamic features of experiments. This direction of exchanges has been beneficial for researchers to set up more naturalistic and well-controlled psychological and neuroscientific experimental studies in the lab^7,10,13,14^. However, the other direction of exchange, i.e., using understandings about psychological and neurocognitive mechanisms of embodied and embedded multisensory perception to guide the design and construction of VR techniques, is also an important, but still a very much neglected task. Results from this study along with previous findings from the research on human perception underscore the power of contextual expectations. We perceive what we expect to experience, be it in reality or in VR. Thus, besides focusing on improving engineering solutions for sensor/actuator technologies and software, the effects of contextual expectations on multisensory processing at the perceptual and brain level are other avenues that can be leveraged to optimize VR technologies.

## Methods

### Participants

Forty-three healthy young adults (24 males, mean age = 23.86 years, range: 18–30 years) participated in the study. Thirty-eight participants were right-handed as measured by the Edinburgh handedness Inventory^62^. All participants provided informed consent before the study and were compensated for their participation. The study was approved by the Ethics Committee of the Technische Universität Dresden (SR-EK-5012021).

### Multimodal stimuli

The stimuli were chosen to represent naturalistic vibration exposure in daily car riding situations. Therefore, four scenes of a common vehicle moving through different road surfaces (cobblestone, fine cobblestone, tarmac, highway) were recorded at a speed of 50 kph from the perspective of front seat. The vertical seat vibrations were recorded with a seat pad accelerometer (B&K 4515B) and low frequency vibrations with a Kistler 8305B10 sensor. Videos were recorded with a Canon EOS 600D Camera with an optical image stabilizer lens. Sound effects at the ear of the driver/passenger were recorded with two B&K 2671 microphones attached to the head rest of the seat.

All virtual multisensory scenarios used in the experiment were based on these multimodal scene recordings. The audio-visual recordings originally recorded from the scenes were used, whereas synthesized vibrations (sinusoidal, amplitude modulated sinusoidal, bandlimited white gaussian noise) were utilized according to previous results^33^. The synthesized vibrations^63^ had been previously rated by human users to be of equal plausibility as the recorded vibrations. It is known that vibration levels affect perceived plausibility of audio-visual scenes^64^. Thus, for each road scene, the acceleration level of the synthesized vibrations was set to 10, 13.25, 16.50, 19.75, 23, 26.25, 29.50, 32.75, and 36 dB in sensation level (i.e., relative to the perceptual threshold of whole-body vibration^65^) for the subjective rating phase of the study. The maximum acceleration level was constrained by the reproduction system, while the minimum acceleration level was chosen to be clearly perceivable (i.e., above the perceptual threshold). Table 1 shows only the parameters of the synthetized vibrations at different sensation levels used for the virtual scenarios of the main experimental task.

**Table 1:** Parameters of the synthesized vibrations at different sensation levels (parameters which are undefined for the respective signal type are marked by “NA”).

Road scenes	Sensation level	(Centre-) frequency	Bandwidth	Modulation frequency
Cobblestone	36 dB	26	50	0
Fine cobblestone	26.25 dB	9	NA	NA
Tarmac	13.25 dB	155	NA	15
Highway	10 dB	7	NA	2

### Experimental design

We experimentally manipulated the plausibility of vibrotactile stimulations in virtual multisensory road scenes by crossing the vibration levels with the four road scenes of different surface smoothness (the audio-visual scenes of the four virtual scenarios are available in the link provided in the “Data availability” statement). Table 2 shows the levels of the vibrotactile stimulations in the high and low plausibility scenarios for the four road scenes in the main experimental task. The main experimental task thus had a 4 ×2 (Scene × Plausibility) within-subject design. During the main experiment, 15 repetitions of each of the eight plausibility by scene combinations were presented and the participants were instructed to passively perceive the multisensory information in the virtual scenario and reflect about the plausibility of the experienced vibration from the car seat. No explicit response was required.

**Table 2:** Vibration levels of the vehicle seat in high plausibility and low plausibility scenarios in the main experimental task for each of the four scenes.

Road scenes	High plausibility	Low plausibility
Cobblestone	36 dB	10 dB
Fine Cobblestone	26.25 dB	10 dB
Tarmac	13.25 dB	36 dB
Highway	10 dB	36 dB

Aside from the main experimental phase, there was a separate phase of subjective rating. During the rating phase, participants were asked to explicitly rate the plausibility of the vibrotactile stimulations across 9 intensity levels (10, 13.25, 16.50, 19.7, 23, 26.25, 29.50, 32.75, and 36 dB) that were paired only once with each of the four road scenes, resulting in 36 scenarios for ratings. The broad range of vibration levels was used for the rating phase to allow assessments of incremental changes in the subjective rating. The participants provided verbal ratings on a quasi-continuous Rohrmann scale with possible values from 0 to 100^33,63,64,66^ with equidistant verbal anchors at 0 (“not at all” plausible), 25 (“slightly” plausible), 50 (“moderately” plausible), 75 (“very” plausible) and 100 (“extremely” plausible). The subjective ratings validated the anticipated difference between the high and low plausibility condition.

### Experimental Setup

The audio-visual-tactile stimuli described in the previous section were presented as multimodal virtual scenarios in the multimodal measurement laboratory described in a previous study^67^. Optical reproduction was achieved with a Full-HD Projector on a screen with a diagonal of 300 cm at the distance of 340 cm to the participants. The acoustic reproduction was realized with a wavefield synthesis system consisting of 464 individually controllable speakers. Such a system recreates the wavefield originally produced by a recorded sound source. Compared to headphone reproduction, it does not have shortcomings such as head localization of sounds which could potentially break immersion. Furthermore, it does not rely on the phantom source effect as utilized in stereo setups. Thus, the audio reproduction of road scene recordings is insensitive to unwanted changes in perceived direction of sounds due to head movements. Finally, vibrations of the seat were reproduced by two systems to cover all the perceivable frequency range of everyday life vibrations spanning from 1 to 500 Hz. Low-frequency vibrations below 15 Hz was presented with a hydraulic motion platform and high- frequency vibration with an electrodynamic shaker attached to the surface of the seat. Due to the current study’s focus on vibration, reproduction was calibrated for each participate to take into account individual differences in weight or body height. The transfer function for each participant was measured prior to the experiment. Subsequently, it was compensated with an FIR filter^68^ to ensure identical vibration reproduction for each participant. The multimodal reproduction system used as the virtual environment in our experiment is shown in the figure (Fig. 1a, b).

### Study procedure

All participants had normal or corrected-to-normal vision and handedness was assessed using the Edinburgh Handedness Inventory^62^. Before the study, two commonly used psychometric tests, i.e., the Identical Pictures Test^69^ and the Spot-the-Word Test^70^ were used to assess participants’ basic cognitive speed and verbal ability. Afterwards, all participants underwent the main experimental phase and the subjective rating phase. The order of these two parts of the study were counterbalanced across participants. In the main experiment, an event-related study design was used to measure brain hemodynamic responses using fNIRS. Participants passively experienced the multisensory virtual scenarios of car riding on four difference road surfaces with concurrent vibrotactile stimulations (vibrations of the seat) of different intensities (see Table 2). Each of the eight virtual scenarios was displayed for a duration of 4 s, while the inter-trial interval (ITI) was jittered according to the following formula:

1 $${{{{{\rm{ITI}}}}}}={T}_{{{{{{\rm{Load}}}}}}}+{T}_{{{{{{\rm{Scene}}}}}}}+{T}_{{{{{{\rm{Transition}}}}}}({{{{{\rm{pseudorandom}}}}}})}+{T}_{{{{{{\rm{Random}}}}}}({{{{{\rm{Geometric}}}}}})}.$$

In this equation *T*_Load_ refers to the time it takes for a given scene to load, *T*_Scene_ refers to the duration of the scene, *T*_Transition_ refers to the time taken to progress to the next stimulus and *T*_Random(Geometric)_ refers to a random number generated according to a geometric distribution. The mean inter-trial interval (ITI) was 14.60 s (ranging from 13.65 to 17.08 s). Each of the 8 virtual scenarios (Table 2) was presented for 15 times, amounting to a total of 120 trials. The presentation order of virtual scenarios was randomized for each participant.

### fNIRS data acquisition

The concentrations of oxygenated and deoxygenated haemoglobin (HbO and HbR, respectively) were collected using the continuous-wave, battery-operated fNIRS system NIRSport 2 (NIRx Medical Technologies, LLC, USA). This system employs two distinct wavelengths (i.e., 760 and 850 nm) with a sampling rate of 4.98 Hz. The NIRS probe was designed using fNIRS Optodes Location Decider (fOLD v2.2^71^), which is a MATLAB-based toolbox that computes optimal optode placement in the 10-10 system in relation to specific brain areas. The Brodmann anatomical atlas was used to localize the optodes to brain regions corresponding to the dorsolateral prefrontal cortex (BA 9), premotor and supplementary motor cortex (BA 6), primary somatosensory cortex (BA 1, BA 2, BA 3), and primary motor cortex (BA 4). For coverage of these areas, the source positions were located at positions AF3, AF4, F3, F2, F4, FC5, FC1, FC2, FC6, C3, Cz, C4, CP1, CP2 while the detector positions were located at AFz, F1, F2, FT7, FC3, FCz, FC4, FT8, C5, C1, C2, C6, CP3, CPz, and CP4 (see Fig. 1c for the probe arrangement). The sensitivity profile of the measurement channels (Fig. 1d) provides visual information as to whether our probe design (montage) is sensitive enough in optical density changes measured by each of the source-detector pairs from changes in the absorption coefficients in the cortex in the regions of interest. As shown in Fig. 1d, our montage resulted in a high sensitivity profile that covers the brain regions of interest.

The fNIRS montage we used consisted of 36 long source-detector separation channels (with inter-optode distance approximately 30 mm with the help of linked optode holders). Of the 36 different channels, 25 channels recorded brain hemodynamic responses from the prefrontal cortex and premotor regions, while the remaining 11 channels recorded activity over the motor and somatosensory cortex. No short-separation channels were used in this study. Since participants would experience whole body vibration, this might lead to artifactual short-distance measurements which could potentially transfer some noise into the signal of interest^72,73^. However, we acknowledge that there may be unwanted physiological confounds, thus, these unwanted physiological confounds potentially due to movement were removed using principal component analysis which has been shown to yield results comparable to short-channel separation techniques^74^ (see below for details on pre-processing).

### fNIRS data pre-processing

Out of the 43 participants who participated in the study, fNIRS data from 4 participants were not obtained due to technical difficulties. Pre-processing was conducted using the Homer3 Toolbox^75^ (BUNPC), while the hemodynamic data was reconstructed using AtlasViewer Toolbox^76^. During pre-processing, bad channels were first identified and excluded using the hmrR_PruneChannels function with the signal-to-noise threshold set to the common criterion of 6.67 (equivalent to coefficient of variation (CV) = 15%; SNR = 1/CV*100). Datasets which contained more than 25% bad channels (i.e., less then CV of 15%^77,78^ were removed from further analysis (*N* = 2). The raw signals of light intensity were converted into changes in optical density using the hmrR_Intensity2OD function. Afterwards, using the hmrR_MotionArtifactbyChannel function, motion artefacts were identified as signal changes in optical density units greater than an amplitude of 0.3 over half a second and marked for one second. Outliers due to motion artefacts that were identified as wavelet coefficients exceeding 1.5 times of the interquartile range were then corrected using the hmrR_MotionCorrectWavelet function^79^. Wavelet filtering is the most effective approach for correcting motion artefacts and it typically reduces motion artefacts up to 93% of cases^80,81^. Trials which still contained motion artefacts that were unsuccessfully corrected were rejected using the hmrR_StimRejection function in the time range covering 2 seconds prior to 4 s post-stimulus. Datasets with more than 20% of rejected trials were removed from the analyses (*N* = 1). Thus, altogether, the effective dataset for analyses of fNIRs data contained data from 36 participants.

Subsequently, a low-pass filter with a cutoff of 0.5 Hz was used to remove high-frequency components (e.g., instrument noise) yet retain brain response of interest^82^. This frequency range, however, may still include physiological artifacts (e.g., those induced by respiration, which is around 0.3 Hz and spontaneous oscillations in arterial blood pressure that is around 0.1 Hz known as the Mayer waves). Since our setup only consisted of long-separation channels, principal component analysis (hmrR_PCAFilter, nSV=1.0) was used to mitigate physiological confounds and interferences from superficial layers of the scalp, skin, and skull^83,84^. In setups without short-separation channels, principal component analysis could yield comparable results as short source-separation channels^74^. In the next step, the pro-processed and filtered optical density was converted into hemodynamic concentration values using hmrR_OD2Conc function based on the modified Beer-Lambert Law. Finally, the hemodynamic response function (HRF) is estimated with a general linear model (GLM) that uses ordinary least squares function^85^. The HRF was modelled with consecutive sequence of Gaussian functions with a standard deviation of 0.5 s and mean separated by 0.5 s (glmSolveMethod= 1, idxBasis=1, paramsBasis = [0.5, 0.5]). The regression time was between −2 to 12 seconds, using the pre-stimulus time of 2 s for baseline correction to account for intra- and inter-individual differences in time-dependent changes in cerebral oxygenation. To account for baseline drift, it was modelled using a third-order polynomial fit^86,87^. For the purpose of further statistical analysis, baseline-corrected mean concentrations of HbO and HbR between 1 to 5 s post-trial onset were extracted. This time window was selected because our stimuli (i.e., the multisensory virtual scenarios) lasted for 4 seconds and to ensure that we include the peak HRF which typically occurs around 4 to 5 s. Since the concentration of HbO has been consistently shown to be more reliable than HbR in reflecting task-induced, event-related cortical responses^50,88–90^, below we only focused on reporting results of HbO concentrations in the main section of this paper.

In order to obtain cortical topography of brain activity, an atlas head model was registered to the participant’s head via digitized points of sources and detectors which allow for a more accurate estimation of the location of brain activation. Image reconstruction of the changes in absorption coefficient in the cortex is possible given experimentally measured changes in optical density and sensitivity profile (i.e., forward matrix)^76^. Briefly, the inverse problem can be solved by inverting the forward matrix. Thus, image reconstruction can be accomplished using the following equation:

2 $${{{{{\bf{x}}}}}}={{{{{{\bf{A}}}}}}}^{{{{{{\rm{T}}}}}}}{({{{{{\bf{A}}}}}}{{{{{{\bf{A}}}}}}}^{{{{{{\rm{T}}}}}}}+\lambda {{{{{\bf{I}}}}}})}^{-1}{{{{{\bf{y}}}}}}.$$

In this equation, **x** refers to the spatial distribution of HbO or HbR absorption perturbation, **A** refers to the forward matrix/sensitivity profile of the registered atlas which is obtained using Monte Carlo photon migration simulation, λ refers to the scalar regularization parameter (in which we used the default value of α = 0.01), **I** refers to the identity matrix while **y** refers to the vector of measurements which is provided as optical density changes.

### Statistics and reproducibility

#### Overall analyses of cortical activities during scenarios of different contextual expectancies

Statistical analysis of the fNIRS data was conducted using Matlab R2018b and RStudio (R 4.1.1). HbO concentration changes was analyzed with linear mixed-effects models using the lme function from the nlme package in R. To investigate if there were any changes in HbO concentration with regards to the different road scenes and plausibility levels, linear mixed-effects models were calculated with maximum likelihood estimation, with Scene (cobblestone, fine cobblestone, tarmac, highway) and Plausibility (low, high) as within-subjects fixed-effects; participants were included as random intercepts with the NIRS channels nested in participants to account for between-subject variability in hemodynamic concentration changes across the channels^78,91–93^.

#### Scene- and region-based analyses

To investigate contextual expectancy modulation of cortical activities during extreme rough or smooth road scenes of high (36 dB) or low (10 dB) levels of vibrotactile stimulation, we then focused on the scenes with the largest difference in sensation level between the most plausible and least plausible scenarios, namely, the Highway and Cobblestone scenes. For each of the two scenes, linear mixed-effects models were analyzed with two regions of interests, i.e., the frontal attentional control and action planning region (dlPFC & premotor) and the sensorimotor region (somatosensory & motor) and Plausibility (High, Low) as fixed-effects and subjects (with channels nested in subjects) as random-effects. To compare the effects of contextual expectancy in modulating perceptual representations of vibrotactile stimulation across scenes, linear mixed-effects models were analyzed using Scene (Highway, Cobblestone) and Intensity (10 dB and 36 dB) as fixed-effects and subjects (with channels nested into subjects) as random effect.

For all the linear mixed-effects models analyses above, the normality of residuals was inspected using Kolmogorov–Smirnov test. Where the residuals were not normally distributed, robust permutation tests were carried out in the following way. Firstly, linear mixed-effects model was analyzed using the lmer function from the lme4 package^94^. If there were convergence issues, the random effects structure was simplified. Permutation tests were conducted on these models using PERMANOVA (number of permutations = 5000) from the ‘predictmeans’ package in R^95^. Since the initial models revealed similar results to the findings from models with the permutation test, we report findings from the initial standard linear mixed-effects models, with factors ‘Scene’ and ‘Plausibility’ as fixed effects, participants as random intercept with NIRS channels nested into participants. For the main effects and interactions, we report partial eta-squared (*η*_*p*_^*2*^*)* for effect size^96,97^ with the following interpretation: *η*_*p*_^*2*^ = 0.01 (small), *η*_*p*_^*2*^ = 0.06 (medium), *η*_*p*_^*2*^ = 0.14 (large). Main effects and interaction effects were followed up by post hoc multiple comparisons with Bonferroni correction using the emmeans package in R^98^. For these post hoc analyses, Adjusted *P*-values are also reported and Cohen’s d are reported as effect size with the following interpretation: *d* = 0.2 (small), *d* = 0.5 (medium), *d* = 0.8 (large).

To account for potential order effects (main experiment phase before rating phase, or the other around), we also included order as a covariate in our analyses; however, using order as a covariate did not change the results. Therefore, we report findings from the original analyses without any covariate.

#### Analyses of frontal-sensorimotor associations

Negative expectancy violation cost was calculated by subtracting HbO concentration in the Highway High Plausibility condition from the HbO concentration in the Cobblestone Low Plausibility condition (i.e., cobblestone_Low Plausibility (10dB)_ − highway_High Plausibility (10dB)_), whereas positive expectancy violation cost was calculated by subtracting HbO concentration in the Cobblestone High Plausibility condition from the HbO concentration in the Highway Low Plausibility condition (highway_Low Plausibility (36dB)_ − Cobblestone_High Plausibility (36dB)_). For the correlational analyses, HbO concentration changes were averaged across all channels in frontal attentional control and actional planning regions (dlPFC & premotor) and in the sensorimotor regions (somatosensory & motor). Outliers that were 3 standard deviations away from the mean were removed to avoid spurious correlations (*N* = 35 for the correlation between negative plausibility violation cost in the frontal and negative plausibility violation cost in the sensorimotor regions (Fig. 4a); *N* = 35 for the correlation between negative plausibility violation cost in the sensorimotor cortex and average HbO concentration in the frontal regions of the high plausibility highway scenario (Fig. 5a); *N* = 35 for the correlation between positive plausibility violation cost in the sensorimotor cortex and average HbO concentration in the frontal regions of the high plausibility cobblestone scenario (Fig. 5b). Correlational analyses were conducted using Pearson’s correlation. Where the data was not normally distributed as indicated by Shapiro–Wilk test of normality, Spearman’s correlation was used.

#### Subjective plausibility ratings of vibration levels

The participants’ subjective ratings were collected across 9 vibration levels (10, 13.25, 16.50, 19.75, 23, 26.25, 29.50, 32.75, and 36 dB) that were each paired once with each of the four road scenes. The ratings showed that indeed across the four scenes participants felt the vibrotactile stimulations to be more plausible in the scenarios experimentally defined to be of high plausibility (mean ratings ranged from 59.93 to 79.12) than in the low-plausibility scenarios (mean ratings ranged from 4.46 to 56.05). The plausibility ratio for each scene was calculated by dividing the difference between the high and low plausibility ratings for that scene by the low plausibility ratings. Because the ratings ranged from 0 to 100, a value of 1 was added to the denominator. Thus, the plausibility ratings ratio was calculated as follows: (Rating_high_ − Rating_low_)/(Rating_low_ + 1). Outliers that were 3 standard deviations away from the mean were removed. To validate as to whether our highly plausible scenes were indeed perceived to be more plausible than the least plausible scenes, we used linear mixed-effects models to analyze participants’ plausibility ratings data with Scene (Cobblestone, Fine Cobblestone, Tarmac, Highway) and Plausibility (Low, High) as within-subjects fixed-effects and subjects as random-effects. Similar to above, where residuals were not normally distributed, ratings data were analyzed using robust permutation tests.

### Reporting summary

Further information on research design is available in the Nature Portfolio Reporting Summary linked to this article.

## Supplementary information

Reporting Summary

## Data Availability

The anonymised data analyzed in this paper and stimuli are available at the following Open Science Framework repository link: https://osf.io/wpn6e/?view_only=cbcd4489f4b847a6a0fd642ef999f3e2.

## Code availability

The code used for analyzing the data is available at the Open Science Framework repository link: https://osf.io/wpn6e/?view_only=cbcd4489f4b847a6a0fd642ef999f3e2.
