# Supplementary material for: Congruence-based contextual plausibility modulates cortical activity during vibrotactile perception in virtual multisensory environments
Source: Commun Biol. 2022 Dec 12;5:1360. doi: 10.1038/s42003-022-04318-4 (PMC9744907; doi:10.1038/s42003-022-04318-4)
Supplement: Supplementary file 1 — Reporting Summary [file 42003_2022_4318_MOESM1_ESM.pdf]

nature portfolio

Corresponding author(s): Kathleen Kang, Shu-Chen Li

Last updated by author(s): Nov 10, 2022

Reporting Summary

Nature Portfolio wishes to improve the reproducibility of the work that we publish. This form provides structure for consistency and transparency in reporting. For further information on Nature Portfolio policies, see our Editorial Policies and the Editorial Policy Checklist.

Statistics

For all statistical analyses, confirm that the following items are present in the figure legend, table legend, main text, or Methods section.

n/a	Confirmed	
☐	☒	The exact sample size (*n*) for each experimental group/condition, given as a discrete number and unit of measurement
☐	☒	A statement on whether measurements were taken from distinct samples or whether the same sample was measured repeatedly
☐	☒	The statistical test(s) used AND whether they are one- or two-sided<br>*Only common tests should be described solely by name; describe more complex techniques in the Methods section.*
☐	☒	A description of all covariates tested
☐	☒	A description of any assumptions or corrections, such as tests of normality and adjustment for multiple comparisons
☐	☒	A full description of the statistical parameters including central tendency (e.g. means) or other basic estimates (e.g. regression coefficient) AND variation (e.g. standard deviation) or associated estimates of uncertainty (e.g. confidence intervals)
☐	☒	For null hypothesis testing, the test statistic (e.g. *F*, *t*, *r*) with confidence intervals, effect sizes, degrees of freedom and *P* value noted<br>*Give P values as exact values whenever suitable.*
☒	☐	For Bayesian analysis, information on the choice of priors and Markov chain Monte Carlo settings
☒	☐	For hierarchical and complex designs, identification of the appropriate level for tests and full reporting of outcomes
☐	☒	Estimates of effect sizes (e.g. Cohen's *d*, Pearson's *r*), indicating how they were calculated

Our web collection on statistics for biologists contains articles on many of the points above.

Software and code

Policy information about availability of computer code

Data collection	Functional Near Infrared Spectroscopy (fNIRS) data was measured using NIRSport2 (NIRx Medical Technologies) and acquired using the Aurora data acquisition software.
Data analysis	fNIRS data was preprocessed in Homer3 and AtlasViewer toolboxes in MATLAB R2018b. Then, statistical analyses were performed using RStudio 4.1.1

For manuscripts utilizing custom algorithms or software that are central to the research but not yet described in published literature, software must be made available to editors and reviewers. We strongly encourage code deposition in a community repository (e.g. GitHub). See the Nature Portfolio guidelines for submitting code & software for further information.

Data

Policy information about availability of data

All manuscripts must include a data availability statement. This statement should provide the following information, where applicable:

- Accession codes, unique identifiers, or web links for publicly available datasets
- A description of any restrictions on data availability
- For clinical datasets or third party data, please ensure that the statement adheres to our policy

All data relevant to the analyses in this manuscript are available at the Open Science Framework repository (https://osf.io/wpn6e/?view_only=cbcd4489f4b847a6a0fd642ef999f3e2) for research purposes only.

Human research participants

Policy information about studies involving human research participants and Sex and Gender in Research.

Reporting on sex and gender	The participants in this study consisted of 24 males and 43 females. We did not collect any gender-based information. We also did not conduct any sex-based analyses because it was not relevant to our study aims.
Population characteristics	43 younger adults (24 males, mean age = 23.86 years, range: 18-30 years) were recruited from Technische Universität Dresden.
Recruitment	Participants were recruited from the existing participant database at the Chair of Lifespan Developmental Neuroscience, Technische Universität Dresden
Ethics oversight	Technische Universität Dresden (EK-5012021)

Note that full information on the approval of the study protocol must also be provided in the manuscript.

Field-specific reporting

Please select the one below that is the best fit for your research. If you are not sure, read the appropriate sections before making your selection.

☒ Life sciences ☐ Behavioural & social sciences ☐ Ecological, evolutionary & environmental sciences

For a reference copy of the document with all sections, see nature.com/documents/nr-reporting-summary-flat.pdf

Life sciences study design

All studies must disclose on these points even when the disclosure is negative.

Sample size	Sample size calculation was performed using G*Power. Based on a repeated-measures ANOVA design, with alpha = 0.05, power = 0.80, a sample size of 40 participants is sufficient to achieve a small effect size (Cohen's f=0.15).
Data exclusions	Out of 43 participants, the fNIRS data were not available for 4 participants due to technical difficulties. During pre-processing, 2 datasets were excluded because there were too many (>25%) bad channels, and 1 dataset was excluded because too many stimuli were rejected.
Replication	Although the study itself was not replicated, the raw data was pre-processed and analysed again in an attempt to reproduce the results found in the manuscript. All results reported in the manuscript were reproduced successfully.
Randomization	Randomization was not relevant because it was a within-subject design.
Blinding	Blinding was not relevant in this case as the study does not involve manipulations which needs to be blinded for the participants or the experimenters.

Reporting for specific materials, systems and methods

We require information from authors about some types of materials, experimental systems and methods used in many studies. Here, indicate whether each material, system or method listed is relevant to your study. If you are not sure if a list item applies to your research, read the appropriate section before selecting a response.

Materials & experimental systems

n/a	Involved in the study	
☒	☐	Antibodies
☒	☐	Eukaryotic cell lines
☒	☐	Palaeontology and archaeology
☒	☐	Animals and other organisms
☒	☐	Clinical data
☒	☐	Dual use research of concern

Methods

n/a	Involved in the study	
☒	☐	ChIP-seq
☒	☐	Flow cytometry
☒	☐	MRI-based neuroimaging
